# Harveian Society

**Published:** 1897-11-06

**Authors:** 


### HARVEIAN SOCIETY.

### Creasote in Phthisis.

At the last meeting of the Harveian Society Dr. Clifford Beale read a paper on "Recent Experience of the Use of Creasote in Phthisis," referring to cases of incipient disease, as well as to the more chronic forms. Beginning with from three to five minims, he had gradually increased the dose until a maximum of about 180 minims a day had been reached. He had used pure beechwood creasote dissolved in cod-liver oil, a method which he found was the least objectionable to the patient. The results had been very satisfactory, and he had found the most marked effects when the larger doses were reached.

The large majority of the patients did not complain of the smell of the drug, and he thought it probable that the creasote was rapidly broken up in the stomach, forming some more stable compound before it was excreted by the kidneys. As the result of his clinical experience he thought that the success which could be obtained from its use was quite equal to, if not better than that which followed the use of guaiacol and its carbonate. Moreover, it was well tolerated by the stomach when dissolved in cod-liver oil, and thus the more irksome and unpleasant methods of administration were unnecessary.

